# Effects of Medial and Lateral Foot Wedge Placement on Lower Limb Biomechanics and Muscle Activation During the Split Squat: A Randomized Crossover Trial

**DOI:** 10.3390/medicina62071249

**Published:** 2026-06-28

**Authors:** Seung Hun Lee, Young Min Lee, Ho Jin Shin, Jung Won Kwon

**Affiliations:** 1 Department of Public Health Sciences, Graduate School, Dankook University, Cheonan 31116, Republic of Korea; tmdgns143@naver.com (S.H.L.); leeyoungmin8259@hanmail.net (Y.M.L.); 2 Wellness Center, Industry-University Collaboration Group, Ansan University, Ansan 15328, Republic of Korea; hojin0911@ansan.ac.kr; 3 Department of Physical Therapy, College of Health and Welfare Sciences, Dankook University, Cheonan 31116, Republic of Korea

**Keywords:** split squat, foot wedge, muscle activation, joint kinematics, ground reaction force, randomized crossover trial

## Abstract

*Background and Objectives*: Foot wedges are widely used to modulate ankle alignment in clinical and athletic settings, yet the effects of mediolateral wedge placement on multi-planar lower limb biomechanics during functional unilateral exercises remain poorly characterized. This study aimed to quantify the effects of medial and lateral foot wedge placement on lower limb joint kinematics, muscle activation, and ground reaction forces (GRFs) during the split squat. *Materials and Methods*: Thirty healthy young adults (12 males, 18 females; 24.5 ± 2.7 years) performed split squats under three randomized conditions using a rigid inclined platform rather than a custom foot orthosis: no wedge (NW), medial wedge (MW), and lateral wedge (LW). Three-dimensional kinematics (Qualisys, 100 Hz), bilateral GRFs (Bertec, 1000 Hz), and surface electromyography (sEMG, 1000 Hz) of the peroneus longus (PL), tibialis anterior (TA), vastus medialis (VM), and vastus lateralis (VL) were recorded synchronously. Repeated-measures ANOVA with Bonferroni post hoc tests and partial eta-squared (*η*^2^*_p_*) were used (α = 0.05). *Results*: LW significantly increased PL and VM activation, sagittal-plane range of motion (ROM) at the ankle, knee, and pelvis, and vertical GRF, compared with MW and NW (*p* < 0.05). MW significantly increased TA and VL activation and reduced sagittal hip ROM (*p* < 0.05). No significant differences were observed for mediolateral or anteroposterior GRF. *Conclusions*: Mediolateral foot wedge placement acutely reorganizes lower limb neuromuscular recruitment, joint kinematics, and vertical ground reaction force during the split squat in healthy young adults. These preliminary findings indicate that wedge orientation, applied via a rigid inclined platform, can acutely and selectively modulate muscle activation patterns; any therapeutic or performance applications, however, were not evaluated here and should not be generalized to conventional clinical orthoses without further investigation.

## 1. Introduction

Closed kinetic chain exercises, such as squats and lunges, are fundamental for enhancing lower limb strength, muscular endurance, and balance, making them staples in both rehabilitation and athletic training [1]. Among these, the split squat is a functional unilateral exercise that requires simultaneous vertical and horizontal force production, offering distinct biomechanical and neuromuscular advantages over traditional bilateral squats [2,3]. Its unilateral nature contributes to improved balance, sprinting performance, and landing stability [4].

Successful execution of the split squat depends on proper alignment of the lower limb kinetic chain, with the ankle joint serving as the primary foundation. Limited ankle dorsiflexion alters sagittal plane knee kinematics and induces compensatory movements in the frontal and transverse planes, increasing the risk of anterior cruciate ligament (ACL) injuries [5,6,7]. Restricted ankle mobility further compromises tibial rotation and pelvic alignment, leading to mechanical inefficiency and potential injury during dynamic weight-bearing tasks [8,9,10]. In high-load tasks such as landing, individuals with limited dorsiflexion exhibit increased knee valgus angles, highlighting the need for ankle-mobility interventions [11].

Because of the mechanical linkage within the lower extremity, ankle position dictates the proximal alignment of the knee and hip. Reduced ankle mobility triggers an ascending kinetic chain reaction, influencing anterior pelvic tilt and lumbar curvature [12,13]. Identifying effective methods to control ankle alignment may therefore provide clinicians with a non-invasive tool to optimize lower limb loading and prevent asymmetric joint loading. Comparative studies between elite and amateur athletes show that professionals exhibit greater ankle range of motion (ROM) and superior neuromuscular control during lunging, underscoring the importance of ankle stability in injury prevention and performance [14,15]. Failure to maintain proper foot–ankle alignment during closed-chain movements may impair muscle coordination and increase injury risks [16].

Foot wedges have been used to modify ankle alignment by modulating eversion and inversion angles, providing afferent proprioceptive input that alters lower limb alignment, muscle activation patterns, and trunk posture [13,17,18]. However, previous studies have largely employed wedges in static postural assessments, and their role in modulating the kinetic and kinematic demands of high-intensity unilateral exercises such as the split squat remains underexplored. Whereas sagittal plane manipulations using heel wedges are well documented, the impact of mediolateral wedge placement on multi-planar kinematics and neuromuscular responses during functional training is poorly understood [5,19,20].

Therefore, this study aimed to quantify the effects of medial and lateral foot wedge placement on lower limb joint kinematics, muscle activation, and ground reaction forces (GRFs) during the split squat. This study examined the effects of mediolateral foot wedge placement on multi-planar lower limb biomechanics during a dynamic split squat task, extending previous work that has primarily focused on sagittal-plane heel wedges [5,17]. It should be noted that foot wedges encompass several distinct categories—including heel wedges that alter sagittal-plane ankle position, medial/lateral forefoot insoles commonly used as clinical orthoses, and rigid inclined platforms such as bamboo yoga blocks—each of which may produce different mechanical effects. The present study employed a rigid bamboo yoga-block wedge rather than a conventional clinical insole; this choice is described in detail in Section 2.4. We hypothesized that foot wedge application would significantly alter lower limb biomechanics compared with the no-wedge condition. Specifically, we expected that lateral wedge placement would enhance vastus medialis activity, increase sagittal plane ROM, and augment vertical GRF, whereas medial wedge placement would shift neuromuscular activation toward the vastus lateralis, reflecting compensatory mechanical adjustments across the lower extremity. The findings are intended to characterize the acute biomechanical and neuromuscular effects of mediolateral wedge placement, thereby providing a mechanistic basis for generating hypotheses about the strategic use of wedges in rehabilitation and strength-training contexts.

## 2. Materials and Methods

### 2.1. Participants

Thirty healthy young adults (12 males, 18 females; 40% male, 60% female) participated in this study. Participants were recruited via departmental bulletin board announcements at Dankook University (Cheonan, Republic of Korea) between June 2024 and August 2024, following approval by the Institutional Review Board (see below). All participants were university students with no formal athletic training and typical, non-athletic levels of recreational physical activity. Inclusion criteria required asymptomatic individuals in their twenties without a history of orthopedic or neurological injuries to the spine or lower limbs within the previous three months. Individuals were excluded if they had foot or ankle instability, prior lower limb surgery, or any visual or vestibular impairments that could affect balance [21]. Eligibility against these criteria was established through self-report and structured screening interview; no specific plantar measurements (e.g., foot posture indices, plantar pressure assessment, or arch-height measurement) were performed prior to data collection, and inclusion was based on the absence of self-reported foot or ankle pathology. All 30 individuals who expressed interest and were screened met the eligibility criteria and were enrolled; no screen failures occurred. The required sample size was determined using G*Power version 3.0.1 (Heinrich-Heine University, Düsseldorf, Germany) based on a preliminary pilot study of five participants. The calculation was performed within the F-test family, using the option “ANOVA: Repeated measures, within factors” to match the present within-subject crossover design with three measurement levels (NW, MW, LW). Input parameters were as follows: effect size f = 0.25 (medium), α = 0.05, statistical power (1 − β) = 0.80, number of groups = 1, number of measurements = 3, correlation among repeated measures = 0.5 (G*Power default), and nonsphericity correction ε = 1. Based on these parameters, the recommended total sample size was 30 participants, which was achieved in the present study. Because the pilot data informed an overall, exploratory effect size rather than a single pre-specified primary endpoint, the resulting sample size should be interpreted as pragmatic rather than fully powered for every individual kinematic, electromyographic, and ground reaction force outcome. The study was conducted in accordance with the Declaration of Helsinki, and the protocol was approved by the Institutional Review Board of Dankook University (approval no. 2024-04-006-003). Written informed consent was obtained from all participants prior to participation.

### 2.2. Experimental Design

**Figure 1:**
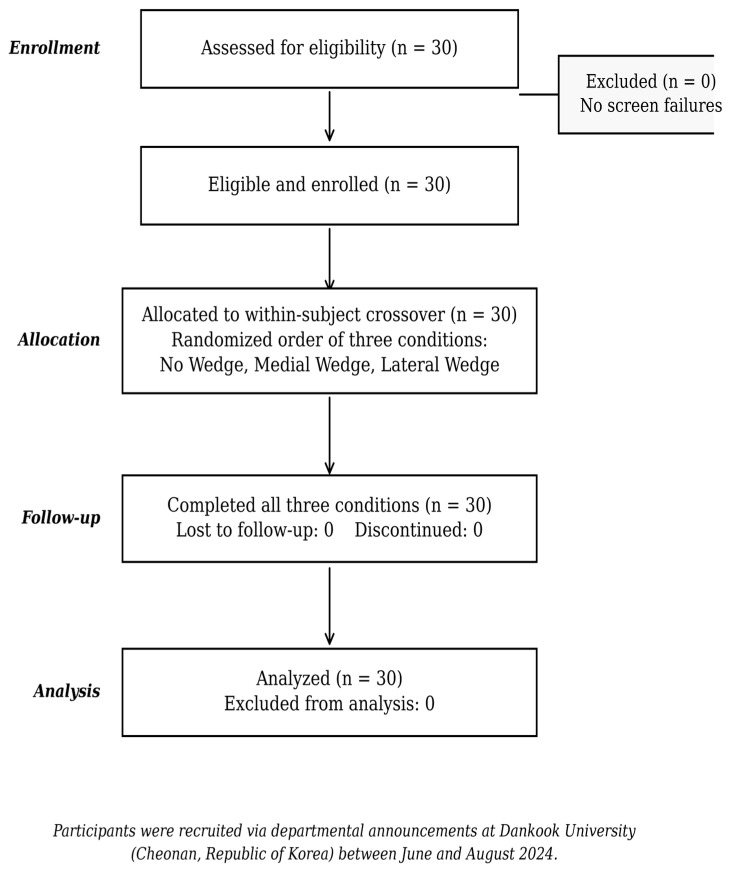
Participant flow through the study, presented according to the CONSORT 2025 guideline. Thirty healthy young adults were assessed for eligibility; all met the inclusion criteria, were enrolled, and completed all three randomized conditions (no wedge, medial wedge, lateral wedge) within a single laboratory session. No participants were excluded, lost to follow-up, or removed from the final analysis.

A randomized, within-subject, three-condition crossover design was conducted in a single laboratory session. This study was a laboratory-based biomechanical experiment in healthy volunteers; the randomized crossover structure was used solely to determine the order of the experimental testing conditions, and no health-related clinical outcomes or therapeutic effects were evaluated. Accordingly, the study does not constitute a clinical trial and was not registered in a clinical trial registry. The three experimental conditions were no wedge (NW), medial wedge (MW), and lateral wedge (LW), applied to the front foot to systematically induce inversion or eversion of the ankle joint. For each participant, the order of the three conditions was independently randomized using a computer-generated random sequence created in Microsoft Excel (Microsoft Corp., Redmond, WA, USA). Although this procedure produced a random allocation, strict balancing across all six possible permutations of the three conditions was not enforced. Prior to data collection, all participants received standardized verbal instructions and a visual demonstration of each wedge condition, and completed familiarization trials of the split squat movement until they could perform the task consistently; familiarization repetitions were not included in subsequent analyses. Allocation concealment was not formally implemented, as participants were necessarily aware of the wedge condition at the moment of testing; however, the testing order was generated and recorded by an investigator and was not disclosed to participants in advance of the laboratory session. Each condition consisted of three repetitions, with a 1 min rest period provided between trials to ensure recovery and minimize muscle fatigue (Figure 1) [22].

### 2.3. Instrumentation

#### 2.3.1. Three-Dimensional Motion Capture

Lower limb joint kinematics were quantified using a three-dimensional motion capture system (Qualisys AB, Göteborg, Sweden) equipped with six infrared cameras at a sampling rate of 100 Hz. Twenty reflective markers were attached according to the Qualisys Biomechanics Marker Set (Figure S1A), including bilateral iliac spines, medial and lateral femoral epicondyles, and malleoli. Specific marker placement is illustrated in Figure S1B. Motion data were processed using Visual 3D software 2021 (C-Motion Inc., Germantown, MD, USA). Movement phases were defined by knee flexion angles: the descent phase began at movement initiation and ended at maximal knee flexion; the ascent phase continued until return to the starting position. Joint angles were calculated in the sagittal, frontal, and transverse planes for the ankle, knee, hip, and pelvis.

#### 2.3.2. Ground Reaction Force

GRF data were collected at 1000 Hz using two Bertec force plates (AM6500; Bertec Corporation, Columbus, OH, USA) positioned under each foot during the split squat and synchronized with the motion capture system. GRFs were recorded in three directions: mediolateral, anteroposterior, and vertical. Positive values in the mediolateral direction indicated medial movement, and positive values in the anteroposterior direction indicated posterior movement. GRF variables were calculated for both the descent and ascent phases. All GRF data were normalized to each participant’s body mass and are reported in N/kg, where one body weight (1 BW) corresponds to approximately 9.81 N/kg [23].

#### 2.3.3. Surface Electromyography

Muscle activation of the vastus medialis (VM), vastus lateralis (VL), tibialis anterior (TA), and peroneus longus (PL) of the forward (non-dominant) leg was recorded using a TeleMyo Desktop DTS system (Noraxon Inc., Scottsdale, AZ, USA) at a sampling frequency of 1000 Hz. Disposable bipolar Ag/AgCl surface electrodes were used, with an inter-electrode distance of 2–3 cm aligned along the longitudinal axis of each muscle belly. Prior to electrode placement, the skin over each recording site was prepared by shaving body hair when present and cleaning the area with alcohol swabs to reduce skin impedance. Electrode placement followed established anatomical guidelines based on the underlying muscle belly, in a manner consistent with SENIAM-style recommendations [24,25], with anatomical landmarks defined as follows: VM, two-thirds of the line between the anterior superior iliac spine (ASIS) and the medial border of the patella, at the most prominent point of the muscle belly; VL, two-thirds of the line between the ASIS and the lateral border of the patella, at the most prominent point of the muscle belly; TA, one-third of the line between the tip of the fibula and the medial malleolus; and PL, 3 cm distal to the head of the fibula. Care was taken during electrode placement and signal inspection to position electrodes over the central portion of each muscle belly along its fiber orientation, in order to minimize potential crosstalk from adjacent muscles. Raw sEMG signals were band-pass filtered at 20–450 Hz and notch-filtered at 60 Hz to remove power-line interference, then full-wave rectified and processed as root-mean-square (RMS) values using Noraxon MyoResearch software (version 1.06). To normalize each signal, maximal voluntary isometric contraction (MVIC) was measured for each muscle. Each MVIC was performed three times for 7 s, with approximately 1–2 min of rest between trials to minimize fatigue, consistent with standard MVIC procedures; the average RMS value of the middle 5 s of each trial was averaged across the three repetitions to obtain a single MVIC reference value per muscle. Manual resistance during MVIC testing was applied by the same trained examiner across all participants to reduce examiner-dependent variability. MVIC test positions were as follows: (1) for VM and VL, participants were seated with the knee flexed at 60° and performed maximal knee extension; (2) for TA, participants were seated with the ankle in a neutral position and performed maximal dorsiflexion; and (3) for PL, participants were seated with the foot in a neutral position and performed maximal eversion against manual resistance. During each split squat trial, the sEMG signal was extracted over the 2 s descent phase and the 2 s ascent phase, processed as the RMS value over each phase, expressed as a percentage of MVIC (%MVIC), and then averaged across the three repetitions of each condition to yield a single participant-level mean per muscle, phase, and condition [26].

### 2.4. Wedge Conditions

Unlike conventional clinical insoles or heel wedges, which are typically soft, custom-fitted, and placed beneath specific regions of the foot, the present study employed a rigid inclined platform to apply a controlled mediolateral inclination across the entire forefoot. Specifically, the experimental conditions were manipulated using a specialized yoga block constructed from bamboo fiber (height: 6 cm; width: 15 cm; length: 30 cm; angle: 8.5°), manufactured by Guangdong Hida Sports (China). This rigid platform was selected to provide a uniform, reproducible angular surface that minimizes inter-participant variability arising from differences in foot anatomy or insole compliance; however, this design choice means that the present intervention is not directly interchangeable with conventional clinical orthoses, and the findings should be interpreted accordingly. To provide a stable and supportive surface tailored to each participant’s foot size, two wedges were combined (Figure S1C). To ensure consistent foot positioning across all conditions, masking tape was used to mark the exact heel position on the wedge. An additional reference line was placed perpendicular to the heel marker to align the distal phalanx of the second toe, ensuring precise multi-planar foot orientation (Figure S1D) [17].

### 2.5. Movement Protocol

All testing was performed barefoot to allow direct contact between the foot and the wedge surface and to enable consistent placement of the foot markers (Figure S1B). Each participant’s dominant leg was identified as the leg most frequently used for kicking, and the split squat was performed with the non-dominant leg positioned forward on the force plate or wedge. The non-dominant limb was selected as the test limb to standardize the side of measurement across participants and to focus on the support function of the front leg, rather than its preferred propulsive function; this choice is consistent with previous unilateral lower-limb studies and is acknowledged as a study choice in the Limitations. Prior to testing, each participant’s functional leg length was measured in the supine position as the distance from the anterior superior iliac spine (ASIS) to the ipsilateral medial malleolus of the non-dominant limb using a flexible tape measure. The split squat step length was then scaled to each participant’s measured leg length so that the longitudinal separation between the front and rear feet was proportionally equivalent across participants of differing stature. The starting position involved standing with the feet positioned approximately pelvis-width apart, the trunk upright, and the arms crossed in front of the chest to minimize upper-body compensation. From this position, participants stepped forward with the non-dominant leg onto the force plate or wedge to the pre-determined, leg-length-scaled step distance. The rear-foot position on the second (rear) force plate was then marked with tape at the heel and at the base of the second distal phalanx so that the same fore–aft and mediolateral foot placement was reproduced across all three conditions (Figure S1D). A metronome set at 60 beats per minute, supplemented by recorded verbal cues at 2 s intervals, was used to standardize the movement tempo at 4 s per repetition [25]. During the descent phase, participants lowered their center of mass over 2 s until the front thigh was approximately parallel to the ground; the parallel position was monitored online by the investigator using visual inspection in conjunction with real-time feedback from the 3D motion capture system (thigh segment orientation relative to the laboratory horizontal), and trials in which the parallel position was not reached were repeated. The ascent phase involved hip and knee extension to return to the starting position over the subsequent 2 s [27]. Detailed verbal instructions and demonstrations were provided, and participants practiced the movement until reaching the required proficiency before data collection. Starting and ending positions of the split squat across the three wedge conditions are illustrated in Figure S2.

### 2.6. Outcome Variables

Outcome variables were categorized a priori by their priority in addressing the research hypotheses. Primary outcomes were defined narrowly to evaluate the central biomechanical question of how mediolateral foot wedge placement modulates lower limb function during the split squat, and comprised: (i) the sagittal-plane range of motion (ROM) of the ankle, knee, and pelvis, (ii) the activation of the peroneus longus (PL), tibialis anterior (TA), vastus medialis (VM), and vastus lateralis (VL) expressed as %MVIC, and (iii) the vertical component of the ground reaction force (vGRF), which directly reflects single-limb load transmission during the squat. All remaining variables—including frontal- and transverse-plane joint ROM, hip-joint sagittal ROM, and the mediolateral and anteroposterior components of the GRF—were treated as secondary, exploratory outcomes intended to provide biomechanical context for the primary findings and to support hypothesis generation for future work.

### 2.7. Statistical Analysis

All analyses were performed with SPSS version 26.0 (IBM Corp., Armonk, NY, USA). Descriptive statistics were used to summarize participants’ general characteristics (age, height, body mass). The unit of analysis was the participant-level mean across the three repetitions performed in each condition; for the electromyographic signals, this mean was computed separately for the descent and ascent phases of each trial before being averaged across repetitions, whereas for kinematic and ground reaction force variables the discrete-point values (peak and corresponding minimum within each phase, used to compute the phase-specific ROM and vGRF) were similarly averaged across the three repetitions. The Shapiro–Wilk test was used to assess the normality of the kinematic, EMG, and GRF data. One-way repeated measures analysis of variance (ANOVA) was performed separately for each outcome variable to compare the three wedge conditions (NW, MW, LW). Mauchly’s test of sphericity was applied to verify the sphericity assumption; when violated, Greenhouse–Geisser corrections were applied. When a significant main effect was detected, pairwise post hoc comparisons (LW vs. MW, LW vs. NW, MW vs. NW) were conducted with Bonferroni correction, yielding an adjusted threshold of α = 0.05/3 ≈ 0.017 for each pairwise contrast within an ANOVA family. Because a large number of repeated-measures ANOVAs were performed across multiple muscles, joints, planes, phases, and force components, the overall analytical framework carries an inflated risk of Type I error that is not fully controlled by within-family Bonferroni correction alone. To address this multiplicity, the analyses were structured hierarchically: primary outcomes (as defined in Section 2.6) were interpreted in a confirmatory manner, whereas secondary outcomes (frontal- and transverse-plane ROM, hip sagittal ROM, and mediolateral and anteroposterior GRF components) were treated as exploratory and hypothesis-generating rather than confirmatory, and should be interpreted with corresponding caution. Effect sizes were computed as partial eta-squared (*η*^2^*_p_*), with values of 0.01, 0.06, and 0.14 interpreted as small, medium, and large effects, respectively. In addition, to support quantitative interpretation of the pairwise contrasts, mean differences and their approximate 95% confidence intervals were computed for the primary outcomes from the reported means and standard deviations using a paired-sample formulation. Because these intervals were reconstructed approximately from summary statistics rather than from the original trial-level output, for which the exact pairwise standard errors were not available, they should be regarded as approximate, and their precision should not be overinterpreted. The level of statistical significance for omnibus tests was set at *p* < 0.05.

## 3. Results

### 3.1. Participant Characteristics

All 30 healthy young adults (12 males, 18 females; 40% male, 60% female) who met the eligibility criteria completed the three experimental conditions, with no dropouts or missing data. All enrolled participants completed all three randomized conditions within a single laboratory session, and data from all 30 participants were included in the final analysis (see Figure 1 for the participant flow diagram). Demographic characteristics were as follows: mean age 24.53 ± 2.70 years, height 165.93 ± 8.91 cm, and body mass 60.78 ± 12.91 kg (Table 1).

### 3.2. Muscle Activation

**Figure 2:**
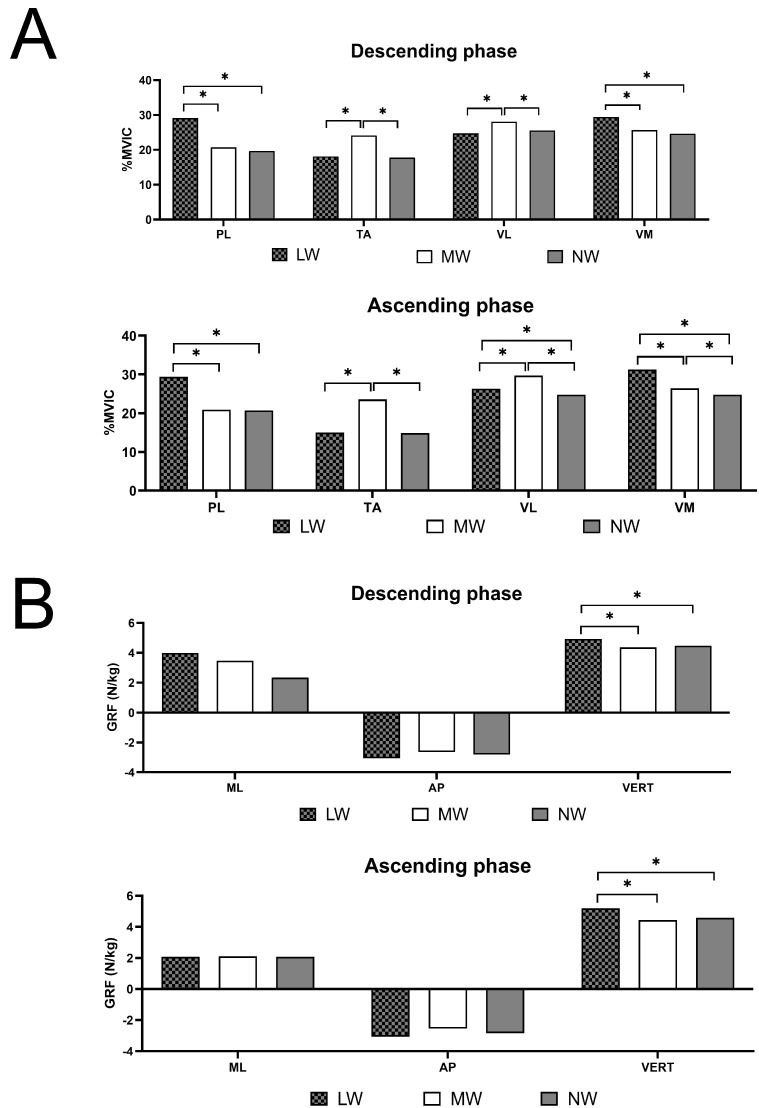
Comparison of kinetic variables according to foot wedge position during the descending and ascending phases of the split squat: (**A**) surface electromyography (sEMG) of the peroneus longus (PL), tibialis anterior (TA), vastus lateralis (VL), and vastus medialis (VM), expressed as a percentage of maximal voluntary isometric contraction (%MVIC); and (**B**) vertical ground reaction force (vGRF), normalized to body mass (N/kg). Bars represent group means; error bars represent ±1 standard deviation. Asterisks (*) indicate statistically significant pairwise differences between the connected conditions based on Bonferroni-adjusted post hoc comparisons following a significant one-way repeated-measures ANOVA (adjusted threshold: α = 0.05/3 ≈ 0.017). LW: lateral wedge; MW: medial wedge; NW: no wedge.

Significant differences were observed in PL, TA, VM, and VL activation across the three wedge conditions (*p* < 0.05; Table 2, Figure 2A). A consistent pattern of muscle recruitment emerged across both movement phases. The LW condition significantly increased PL activation compared with NW during both phases (descending: approximate mean difference [MD] +9.44 %MVIC, 95% confidence interval [CI] [+7.43, +11.45]; ascending: MD +8.68, 95% CI [+6.59, +10.77]) and similarly increased VM activation relative to NW (descending: MD +4.80, 95% CI [+3.47, +6.13]; ascending: MD +6.48, 95% CI [+5.19, +7.77]). Conversely, the MW condition elicited significantly higher TA activation than NW (descending: MD +6.39, 95% CI [+3.63, +9.15]; ascending: MD +8.71, 95% CI [+8.16, +9.26]) and higher VL activation than NW during the ascending phase (MD +4.94, 95% CI [+3.70, +6.18]). These findings indicate that lateral wedging selectively augments PL and VM recruitment, whereas medial wedging shifts muscular demand toward the TA and VL. All pairwise mean differences with 95% CIs are reported in Supplementary Table S1.

### 3.3. Ground Reaction Force

Vertical GRF was significantly affected by wedge placement during both the descent and ascent phases (*p* < 0.05; Table 3, Figure 2B). Post hoc analysis showed that the LW condition produced significantly greater vertical GRF than both the MW and NW conditions, with the largest contrasts observed against MW (descending: approximate MD +0.57 N/kg, 95% CI [+0.36, +0.78]; ascending: MD +0.75 N/kg, 95% CI [+0.56, +0.94]) and consistent, smaller increases relative to NW (descending: MD +0.46 N/kg, 95% CI [+0.24, +0.68]; ascending: MD +0.61 N/kg, 95% CI [+0.38, +0.84]). No significant differences were observed in mediolateral or anteroposterior GRF across any of the wedge conditions, suggesting that the mechanical influence of the wedges was primarily confined to the vertical force component. All pairwise mean differences with 95% CIs for vertical GRF are reported in Supplementary Table S2.

### 3.4. Joint Kinematics

**Figure 3:**
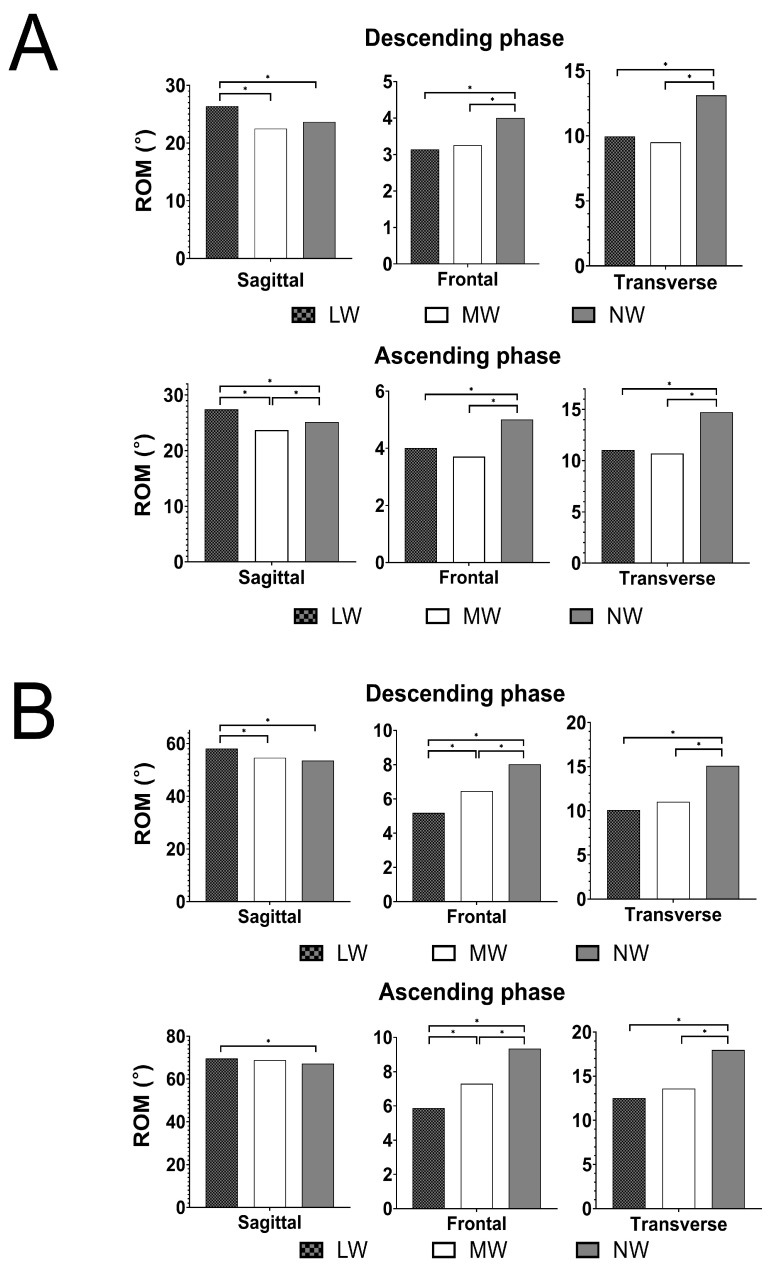
Comparison of joint range of motion (ROM, in degrees) at the (**A**) ankle and (**B**) knee in the sagittal, frontal, and transverse planes according to foot wedge position during the descending and ascending phases of the split squat. Bars represent group means; error bars represent ±1 standard deviation. Asterisks (*) indicate statistically significant pairwise differences between the connected conditions based on Bonferroni-adjusted post hoc comparisons following a significant one-way repeated-measures ANOVA (adjusted threshold: α = 0.05/3 ≈ 0.017). LW: lateral wedge; MW: medial wedge; NW: no wedge.

**Figure 4:**
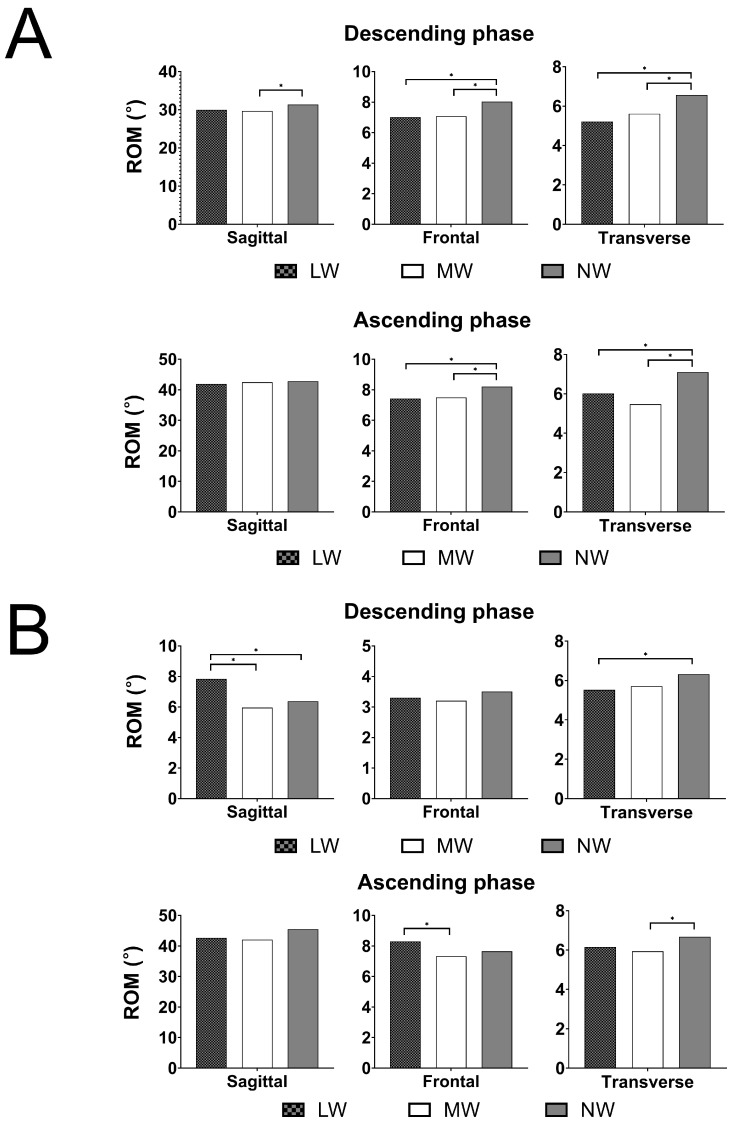
Comparison of joint range of motion (ROM, in degrees) at the (**A**) hip and (**B**) pelvic tilt in the sagittal, frontal, and transverse planes according to foot wedge position during the descending and ascending phases of the split squat. Bars represent group means; error bars represent ±1 standard deviation. Asterisks (*) indicate statistically significant pairwise differences between the connected conditions based on Bonferroni-adjusted post hoc comparisons following a significant one-way repeated-measures ANOVA (adjusted threshold: α = 0.05/3 ≈ 0.017). LW: lateral wedge; MW: medial wedge; NW: no wedge.

Joint ROM differed significantly across all three planes for the ankle, knee, hip, and pelvis (*p* < 0.05; Table 4). In the sagittal plane, the LW condition showed significantly greater ROM than NW and MW at the ankle during both phases (descending vs. MW: approximate MD +3.88°, 95% CI [+2.52, +5.24]; descending vs. NW: MD +2.72°, 95% CI [+1.14, +4.30]; Figure 3A), at the knee during the descent phase (LW vs. MW: MD +3.44°, 95% CI [+0.80, +6.08]; LW vs. NW: MD +4.54°, 95% CI [+1.95, +7.13]; Figure 3B), and in pelvic tilt during the descent phase (LW vs. MW: MD +1.88°, 95% CI [+1.19, +2.57]; LW vs. NW: MD +1.46°, 95% CI [+0.78, +2.14]; Figure 4B). In contrast, ascending sagittal hip ROM did not differ significantly across conditions (Figure 4A; see Discussion Section 4.2 for further interpretation). In the frontal and transverse planes, both MW and LW resulted in significantly lower ROM at the ankle, knee, and hip compared with NW (*p* < 0.05). All pairwise mean differences with 95% CIs for the primary sagittal-plane outcomes are reported in Supplementary Table S3.

## 4. Discussion

This study investigated how variations in foot wedge placement modulate lower limb muscle activation, joint kinematics, and ground reaction forces during the split squat. The findings demonstrate that wedge positioning systematically reorganizes the kinetic chain, indicating that distal modifications can strategically alter neuromuscular demand and mechanical loading patterns throughout the lower extremity.

### 4.1. Neuromuscular Adaptations

Mechanistically, the wedge-dependent shifts in distal muscle activity are consistent with the established roles of the PL and TA as primary dynamic stabilizers of the ankle-foot complex: the PL controls excessive subtalar inversion and contributes to lateral ankle stability during single-limb loading, whereas the TA decelerates plantarflexion and resists excessive eversion of the rearfoot [28,29]. Placing the wedge laterally promotes a relative eversion of the supported foot and is therefore expected to increase PL demand, whereas a medial wedge induces relative inversion and increases TA demand. Beyond changes in joint position, these activation shifts are also likely reinforced by alterations in muscle–tendon unit length; eccentric contractions performed at varied muscle lengths are known to significantly affect both force production and injury risk [30,31].

Wedge placement also modulated quadriceps recruitment in a mediolateral-specific manner. Rather than reflecting a generic increase in “medial and lateral stabilizers,” these shifts can be more specifically attributed to compensatory adjustments of the medial and lateral quadriceps in response to the frontal-plane knee loading imposed by the wedge: lateral wedging tends to reduce relative knee-valgus loading and increase demand on the VM, which contributes to medial tracking of the patella, whereas medial wedging tends to increase relative knee-varus loading and place greater demand on the VL [32,33,34]. This interpretation is consistent with prior research on the synergistic role of the medial and lateral quadriceps in stabilizing the patellofemoral joint in response to frontal-plane perturbations. It should be emphasized, however, that the four muscles examined here represent only a subset of the musculature involved in the split squat; major contributors to lower-limb stabilization, such as the gluteus maximus, gluteus medius, gastrocnemius, and soleus, were not recorded. The neuromuscular patterns described therefore constitute a partial characterization of the muscular strategies underlying the task rather than a complete account.

### 4.2. Joint Kinematics

The lateral wedge increased sagittal-plane ROM in the ankle, knee, and pelvis compared with the other conditions. These changes may reflect enhanced pronation and dorsiflexion facilitated by the oblique axis mechanics of the subtalar joint, which promotes greater exercise depth and improved vertical alignment [35]. Increased dorsiflexion contributes to greater tibial inclination, allowing for a more stable and deeper squat while reducing compensatory trunk movements and frontal-plane stress at the knee [12,17]. The hip joint, in contrast, showed a more nuanced kinematic pattern. Sagittal hip ROM differed significantly across conditions during the descent phase (Table 4), but did not differ significantly during the ascent phase; frontal- and transverse-plane hip ROM were reduced with wedge use in both phases compared with NW. Thus, while wedge use was associated with subtle, plane-specific changes in hip kinematics, ascending sagittal hip ROM was preserved across conditions and showed only a non-significant decreasing trend. These responses likely reflect compensatory trunk adjustments aimed at maintaining the standardized ‘thigh-parallel-to-ground’ position despite the increased ankle contribution. As frontal- and transverse-plane joint motion and hip sagittal ROM were pre-specified as secondary, exploratory outcomes, the associated findings should be regarded as hypothesis-generating rather than confirmatory and interpreted with caution accordingly. Similar reductions in hip flexion have been observed when ankle dorsiflexion increases during lunging tasks [36]. Beyond their statistical patterns, the joint ROM data provide insight that complements the EMG findings: whereas %MVIC quantifies neuromuscular demand at the muscle level, sagittal-plane ROM at the ankle, knee, and pelvis quantifies how that neuromuscular demand translates into actual joint excursion and movement quality. Observing neuromuscular demand and joint excursion concurrently under controlled wedge conditions may help generate mechanistic hypotheses about how distal foot positioning relates to each dimension. Because no rehabilitation, functional, or clinical outcomes were assessed, and these patterns were obtained in asymptomatic young adults, any relevance to rehabilitation protocols or exercise progression remains hypothetical and would require direct evaluation in clinical populations with relevant impairments.

### 4.3. Kinetic Efficiency

Vertical GRF was significantly higher in the LW condition than in the MW and NW conditions. This finding is consistent with the observed increases in PL and VM activation and greater sagittal-plane ankle ROM, which together enhance force-transmission efficiency and reduce compensatory instability [14,37]. The lack of significant differences in mediolateral or anteroposterior GRF may be attributed to the high level of joint control in our healthy participants. As reported previously, wedge-induced kinetic asymmetries are typically more pronounced in populations with structural deformities or knee osteoarthritis [38].

The practical significance of these effects should be interpreted with care. While several outcomes showed large effect sizes—most notably the changes in muscle activation and in frontal- and transverse-plane joint control—others corresponded to comparatively small absolute differences. For example, the wedge-related changes in pelvic sagittal ROM (on the order of 1–2°) and in vertical GRF (below 0.6 N/kg) were statistically significant yet modest in magnitude, and their biomechanical relevance in real-world training or clinical settings remains uncertain. Statistical significance alone should therefore not be equated with practical relevance, and the smaller effects in particular warrant confirmation before being assigned functional importance. Conversely, a number of outcomes—most notably tibialis anterior activity during the ascending phase and several frontal-plane kinematic variables—yielded exceptionally large effect sizes. These magnitudes should be interpreted in light of the study conditions: the relatively homogeneous sample of healthy young adults, the standardized and tightly controlled laboratory protocol, and the within-subject design all reduce inter-individual variability and may consequently inflate the apparent effect sizes relative to those expected in more heterogeneous populations or less constrained, real-world settings. The large effects observed here should therefore be regarded as condition-specific rather than as direct estimates of the magnitude likely to occur in applied contexts. For the same reason, and because the reported confidence intervals were reconstructed approximately from summary statistics, the precision of these estimates should not be overinterpreted.

### 4.4. Clinical and Practical Applications

From a mechanistic perspective, the principal contribution of this study is the demonstration that the mediolateral position of a simple foot wedge can be used to selectively bias neuromuscular demand within the lower-limb kinetic chain: lateral wedging acutely increased PL and VM activation and sagittal-plane mobility at the ankle, knee, and pelvis, whereas medial wedging acutely shifted recruitment toward the TA and VL. Rather than uniformly increasing overall muscle activity, wedge placement redirected activation toward specific target muscles in a predictable, position-dependent manner, providing a quantitative basis for using wedge orientation as a deliberate tool for selective muscle activation during a functional closed-chain exercise.

Two specific contexts illustrate how this principle might be translated into practice. In a rehabilitation context, chronic lateral ankle instability is characterized by diminished peroneal activation and a consequently elevated risk of recurrent sprains; because lateral wedging selectively increased PL activation during a weight-bearing, closed-chain task that resembles daily and athletic movements, it generates the testable hypothesis that wedge-augmented split squats could complement conventional open-chain peroneal strengthening by recruiting the PL under functional loading conditions. In an injury-prevention context for jumping and cutting sports, an unfavorable vastus medialis-to-vastus lateralis activation balance is associated with poorer frontal-plane knee control; because lateral wedging selectively increased VM activation, it generates the testable hypothesis that incorporating wedge orientation into squat-based training could help bias the VM:VL balance in a direction relevant to knee-injury-prevention programs. In both cases, these acute biomechanical responses were observed in asymptomatic young adults and therefore represent mechanistic hypotheses rather than demonstrated clinical effects. Any clinical or rehabilitative implications consequently remain speculative until confirmed in the relevant patient and athlete populations, which were not assessed in the present study and require direct evaluation.

Building on the observed muscle-activation patterns, several extensions of this paradigm warrant systematic investigation. First, the split squat in the present study was performed without external resistance; introducing graded external loads (e.g., a percentage of body mass through dumbbells or a barbell) would test whether the wedge-induced activation shifts in PL, TA, VM, and VL are amplified, attenuated, or maintained as joint loading increases. Second, applying the mediolateral wedge paradigm to other unilateral functional exercises (such as forward lunges, lateral lunges, or step-up tasks) would test whether the observed neuromuscular reorganization is specific to the split squat or generalizes across closed-kinetic-chain unilateral movements. Third, varying squat depth or stance length would test whether the wedge effect is robust across a wider range of biomechanical demands. Together, these systematic extensions would clarify the boundary conditions under which mediolateral foot wedging produces meaningful neuromuscular modulation.

### 4.5. Limitations

Several limitations should be acknowledged. First, regarding study design, the cross-sectional nature of the present work and the absence of external loading preclude inferences about long-term adaptations, and phase-specific activation trends were not consistently observed—likely owing to the standardized movement tempo and unloaded condition, as phase-specific differences are typically more apparent under explosive or externally loaded conditions [5,39]. In addition, the intervention employed a rigid inclined platform rather than a custom-made wedge or insole, so the present results should not be extrapolated directly to conventional clinical orthoses, whose material properties and contact characteristics differ. Second, regarding the participant sample, recruitment was confined to healthy young adults, eligibility relied on self-report, and habitual physical activity, plantar morphology (e.g., arch type, foot posture index, plantar pressure), and static lower-limb alignment (e.g., varus/valgus alignment, femoral or tibial torsion, rearfoot position) were not characterized. In addition, a single standardized wedge inclination (8.5°) was applied to all participants to ensure reproducibility rather than being individualized, and effects were not stratified by participant characteristics. Because the intervention specifically targets foot and ankle mechanics, these unmeasured factors may substantially influence individual responses and limit the external validity and generalizability of the findings. The within-subject design ensured that these characteristics remained constant across conditions, so their influence is on external rather than internal validity; characterizing them and testing baseline-dependent effect modification through stratified analysis remain essential objectives for future work. Third, regarding measurement scope, only four lower limb muscles were assessed; the activation of other primary stabilizers, such as the gluteus maximus, gluteus medius, gastrocnemius, and soleus, was not measured, so the observed responses represent only a partial characterization of the muscular strategies involved in the task. In addition, the split squat was performed only with the non-dominant leg forward, so whether the present findings generalize to dominant-leg-forward stances and to populations with marked inter-limb asymmetry remains to be confirmed. Fourth, regarding the analytic approach, only the discrete peak values of kinematic and kinetic variables were analyzed; future work using statistical parametric mapping (SPM) of the full time-series signals may reveal phase-specific differences not captured here. In addition, the large number of repeated-measures comparisons across muscles, joints, planes, and phases increases the overall risk of Type I error; accordingly, the secondary outcomes in particular should be regarded as exploratory and hypothesis-generating rather than confirmatory. Fifth, regarding randomization, although the order of the three conditions was randomized for each participant, strict balancing across all six possible permutations was not enforced, and trial order was not entered as a covariate. Such incomplete counterbalancing leaves open the possibility that learning effects, motor adaptation, or fatigue across the repeated conditions influenced the responses, although familiarization trials before testing and a one-minute rest between conditions were used to mitigate learning and fatigue, respectively. Future studies with larger samples could implement a counterbalanced Latin-square design and formally test for order or carryover effects. Finally, regarding statistical reporting, the exact pairwise post hoc standard errors from the original output files were not retrievable; the 95% confidence intervals for the mean differences in Table 2, Table 3 and Table 4 were therefore reconstructed using a paired-sample approximation and should be interpreted as descriptive rather than fully calibrated inferential statistics. Future studies should pre-register a small set of primary endpoints, incorporate varied loads and tempos, objective plantar and alignment assessments, and report exact pairwise statistics with standardized effect sizes to support quantitative meta-analytic synthesis.

## 5. Conclusions

In healthy young adults performing unloaded split squats, an 8.5° mediolateral wedge acutely reorganized lower-limb neuromuscular recruitment, joint range of motion, and vertical ground reaction force: lateral wedge placement increased PL and VM activation and sagittal-plane mobility, whereas medial wedge placement recruited the TA and VL. These results provide mechanistic evidence only and do not demonstrate clinical efficacy. Because the intervention used a rigid inclined platform rather than a conventional foot orthosis, and because several secondary outcomes were exploratory and subject to an increased risk of Type I error, the findings should be regarded as hypothesis-generating and should not be extrapolated directly to clinical orthotic practice. Whether these acute changes translate into longer-term clinical benefit in patient populations—such as those with anterior cruciate ligament injury, patellofemoral pain syndrome, or other knee-loading impairments—remains to be determined in future randomized controlled trials that directly assess injury, pain, and functional outcomes.

## Figures and Tables

**Table 1:** General characteristics.

Variable	Mean ± SD (*n* = 30)
Sex (M/F)	12/18
Age (years)	24.53 ± 2.70
Height (cm)	165.93 ± 8.91
Weight (kg)	60.78 ± 12.91

Note. Values represent mean ± standard deviation; M: male; F: female.

**Table 2:** Comparison of Muscle activity according to foot wedge position during split squat exercise.

	Muscles	LW (%MVIC)	MW (%MVIC)	NW (%MVIC)	*F*	*p*	*η* ^2^ *_p_*
Descending phase	PL	29.12 ± 4.24	20.72 ± 6.73	19.68 ± 3.33	52.035	<0.001 *	0.642
	TA	18.16 ± 3.84	24.14 ± 5.37	17.75 ± 5.07	35.268	<0.001 *	0.549
	VL	24.76 ± 1.57	28.07 ± 3.76	25.53 ± 3.23	12.930	<0.001 *	0.308
	VM	29.40 ± 2.70	25.66 ± 2.43	24.60 ± 2.32	118.284	<0.001 *	0.803
Ascending phase	PL	29.39 ± 3.56	20.91 ± 3.97	20.71 ± 4.31	58.044	<0.001 *	0.667
	TA	14.97 ± 1.18	23.55 ± 0.41	14.84 ± 1.41	447.316	<0.001 *	0.939
	VL	26.33 ± 2.68	29.72 ± 2.58	24.78 ± 2.07	41.172	<0.001 *	0.587
	VM	31.25 ± 2.26	26.40 ± 2.08	24.77 ± 2.60	45.326	<0.001 *	0.610

Note. Values represent mean ± standard deviation; LW: lateral wedge; MW: medial wedge; NW: no wedge; PL: peroneus longus; TA: tibialis anterior; VL: vastus lateralis; VM: vastus medialis; *η*^2^*_p_*: partial eta-squared (effect size); * *p* < 0.05. Bonferroni-adjusted threshold for pairwise contrasts: α = 0.017. Approximate mean differences and 95% confidence intervals for all pairwise contrasts of the primary muscles are reported in Supplementary Table S1.

**Table 3:** Comparison of GRF data according to foot wedge position during split squat exercise.

	GRF	LW (N/kg)	MW (N/kg)	NW (N/kg)	*F*	*p*	*η* ^2^ *_p_*
Descending phase	ML	3.98 ± 4.80	3.47 ± 10.83	2.34 ± 15.40	0.231	0.795	0.008
	AP	−3.06 ± 2.11	−2.64 ± 1.96	−2.81 ± 1.66	1.431	0.247	0.047
	VERT	4.93 ± 0.37	4.36 ± 0.41	4.47 ± 0.45	23.937	<0.001 *	0.452
Ascending phase	ML	2.07 ± 6.79	2.10 ± 3.81	2.08 ± 6.15	0.001	0.999	0.000
	AP	−3.07 ± 1.70	−2.55 ± 1.79	−2.85 ± 2.11	2.910	0.062	0.091
	VERT	5.19 ± 0.42	4.44 ± 0.29	4.58 ± 0.44	50.856	<0.001 *	0.637

Note. Values represent mean ± standard deviation; GRF: ground reaction force; ML: mediolateral; AP: anteroposterior; VERT: vertical; LW: lateral wedge; MW: medial wedge; NW: no wedge; *η*^2^*_p_*: partial eta-squared (effect size); * *p* < 0.05. Bonferroni-adjusted threshold for pairwise contrasts: α = 0.017. Approximate mean differences and 95% confidence intervals for the primary vertical GRF contrasts are reported in Supplementary Table S2.

**Table 4:** Comparison of joint ROM according to foot wedge position during split squat exercise.

	Phase	Plane	LW (Degrees)	MW (Degrees)	NW (Degrees)	*F*	*p*	*η* ^2^ *_p_*
Ankle	Descending	Sagittal	26.38 ± 3.18	22.50 ± 1.75	23.66 ± 2.81	23.425	<0.001 *	0.447
		Frontal	3.14 ± 0.59	3.25 ± 0.60	4.00 ± 0.78	14.607	<0.001 *	0.335
		Transverse	9.95 ± 2.26	9.50 ± 1.66	13.10 ± 1.38	39.628	<0.001 *	0.577
	Ascending	Sagittal	27.42 ± 3.02	23.69 ± 2.34	25.17 ± 2.35	25.730	<0.001 *	0.470
		Frontal	4.01 ± 0.60	3.71 ± 0.59	5.01 ± 0.58	39.244	<0.001 *	0.575
		Transverse	11.03 ± 1.84	10.72 ± 1.30	14.71 ± 1.93	58.813	<0.001 *	0.670
Knee	Descending	Sagittal	58.07 ± 5.06	54.63 ± 4.94	53.53 ± 4.76	13.021	<0.001 *	0.310
		Frontal	5.18 ± 0.56	6.46 ± 1.01	8.02 ± 1.06	75.233	<0.001 *	0.722
		Transverse	10.08 ± 0.89	11.03 ± 1.87	15.11 ± 2.33	66.426	<0.001 *	0.696
	Ascending	Sagittal	69.58 ± 4.51	68.85 ± 1.15	67.13 ± 5.41	3.967	0.024 *	0.120
		Frontal	5.87 ± 0.56	7.29 ± 0.61	9.34 ± 0.69	253.965	<0.001 *	0.898
		Transverse	12.52 ± 2.67	13.58 ± 2.68	17.97 ± 2.87	75.425	<0.001 *	0.722
Hip	Descending	Sagittal	29.95 ± 3.77	29.66 ± 1.51	31.32 ± 3.92	3.404	0.040 *	0.105
		Frontal	7.02 ± 1.68	7.07 ± 1.94	8.03 ± 1.49	5.581	0.006 *	0.161
		Transverse	5.21 ± 1.28	5.62 ± 1.26	6.57 ± 1.41	18.549	<0.001 *	0.390
	Ascending	Sagittal	41.88 ± 4.29	42.46 ± 3.99	42.78 ± 5.07	0.813	0.449	0.027
		Frontal	7.42 ± 1.72	7.49 ± 1.45	8.20 ± 1.75	4.173	0.020 *	0.126
		Transverse	6.02 ± 1.26	5.47 ± 0.91	7.10 ± 1.50	18.576	<0.001 *	0.390
Pelvic	Descending	Sagittal	7.83 ± 1.33	5.95 ± 1.30	6.37 ± 1.26	32.479	<0.001 *	0.528
		Frontal	3.30 ± 0.94	3.20 ± 0.77	3.50 ± 0.72	1.696	0.192	0.055
		Transverse	5.52 ± 1.47	5.71 ± 1.26	6.32 ± 1.14	5.337	0.007 *	0.155
	Ascending	Sagittal	42.60 ± 5.59	42.05 ± 7.84	42.51 ± 5.29	0.144	0.866	0.005
		Frontal	8.29 ± 1.77	7.33 ± 1.58	7.65 ± 1.62	5.478	0.007 *	0.159
		Transverse	6.16 ± 1.16	5.94 ± 1.25	6.68 ± 1.13	3.846	0.027 *	0.117

Note. Values represent mean ± standard deviation; ROM: range of motion; LW: lateral wedge; MW: medial wedge; NW: no wedge; *η*^2^*_p_*: partial eta-squared (effect size); * *p* < 0.05. Bonferroni-adjusted threshold for pairwise contrasts: α = 0.017. Approximate mean differences and 95% confidence intervals for the primary sagittal-plane ROM contrasts (ankle, knee, pelvis) are reported in Supplementary Table S3.

## Data Availability

The data presented in this study are available on reasonable request from the corresponding author. The data are not publicly available due to privacy restrictions associated with the IRB approval.

## Supplementary Materials

The following supporting information can be downloaded at: https://www.mdpi.com/article/10.3390/medicina62071249/s1. Figure S1: Detailed experimental apparatus: (A) Qualisys Biomechanics Marker Set illustration; (B) anatomical marker placement (a) anterior view; (b) posterior view; (c) lateral view; (C) 8.5° yoga block wedge design; and (D) Marking of the heel and the base of the second distal phalanx with masking tape for standardized foot orientation; Figure S2: Starting and ending positions of the split squat exercise across different wedge conditions (No Wedge, Medial Wedge, and Lateral Wedge) to illustrate the standardized movement protocol; Table S1. Approximate pairwise mean differences (MD) with 95% confidence intervals (CI, in brackets) for muscle activation (%MVIC) during the descent and ascent phases of the split squat. Negative values indicate that the first condition was lower than the second; Table S2. Approximate pairwise mean differences (MD) with 95% confidence intervals (CI, in brackets) for vertical ground reaction force (N/kg) during the descent and ascent phases of the split squat; Table S3. Approximate pairwise mean differences (MD) with 95% confidence intervals (CI, in brackets) for sagittal-plane range of motion (in degrees) at the ankle, knee, and pelvis during the descent and ascent phases of the split squat.

## Abbreviations

ACL	Anterior cruciate ligament
ANOVA	Analysis of variance
GRF	Ground reaction force
LW	Lateral wedge
MVIC	Maximal voluntary isometric contraction
MW	Medial wedge
NW	No wedge
PL	Peroneus longus
RMS	Root mean square
ROM	Range of motion
sEMG	Surface electromyography
TA	Tibialis anterior
VL	Vastus lateralis
VM	Vastus medialis
