# Supplementary material for: Effects of Medial and Lateral Foot Wedge Placement on Lower Limb Biomechanics and Muscle Activation During the Split Squat: A Randomized Crossover Trial
Source: Medicina (Kaunas). 2026 Jun 28;62(7):1249. doi: 10.3390/medicina62071249 (PMC13413874; doi:10.3390/medicina62071249)
Supplement: Supplementary file 1 [file medicina-62-01249-s001.zip › medicina-4336506-supplementary.pdf]

Figure S1. Detailed experimental apparatus: (A) Qualisys Biomechanics Marker Set illustration; (B) anatomical marker placement (a) anterior view; (b) posterior view; (c) lateral view; (C) 8.5° yoga block wedge design; and (D) Marking of the heel and the base of the second distal phalanx with masking tape for standardized foot orientation.

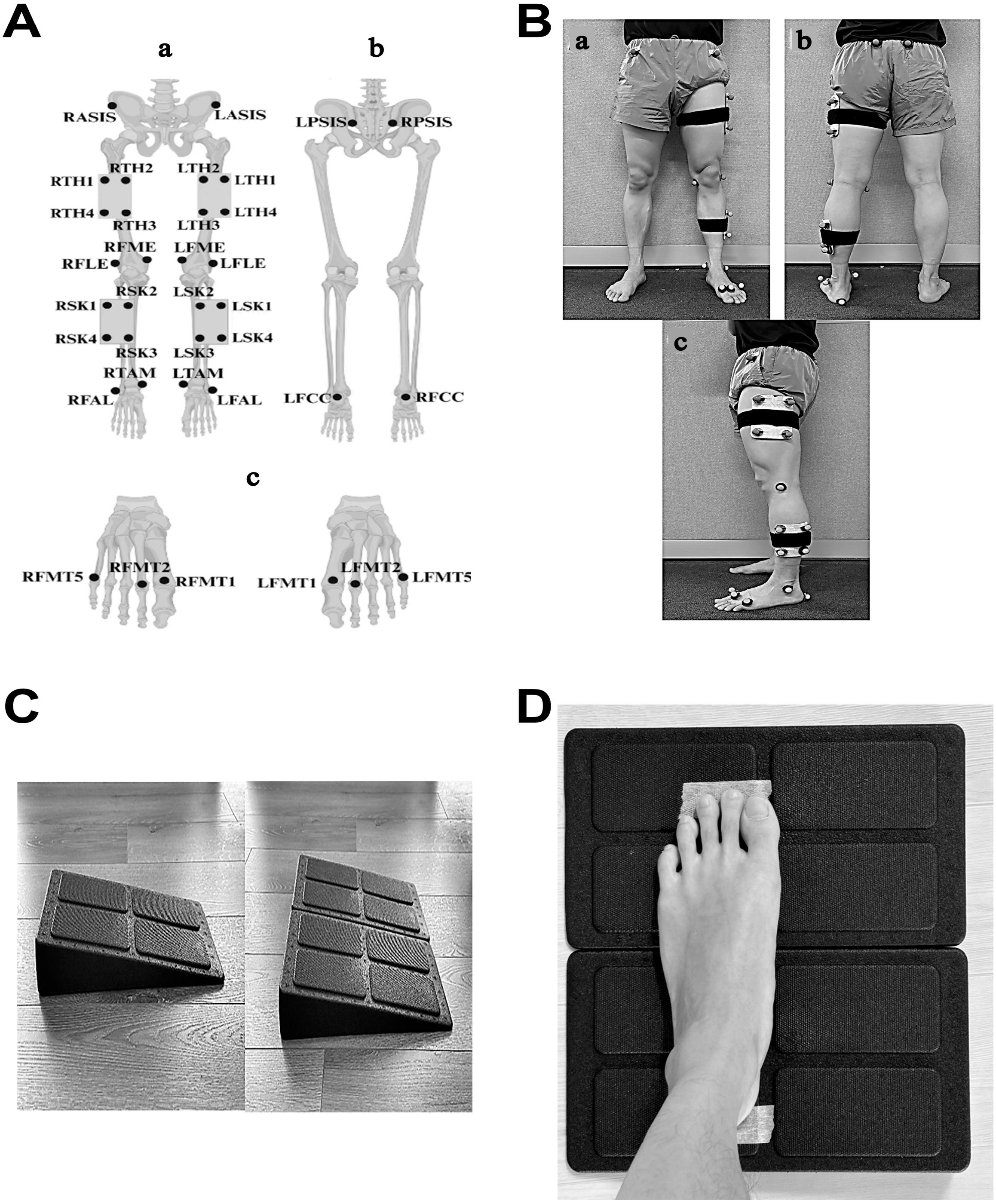

Figure S2. Starting and ending positions of the split squat exercise across different wedge conditions (No Wedge, Medial Wedge, and Lateral Wedge) to illustrate the standardized movement protocol.

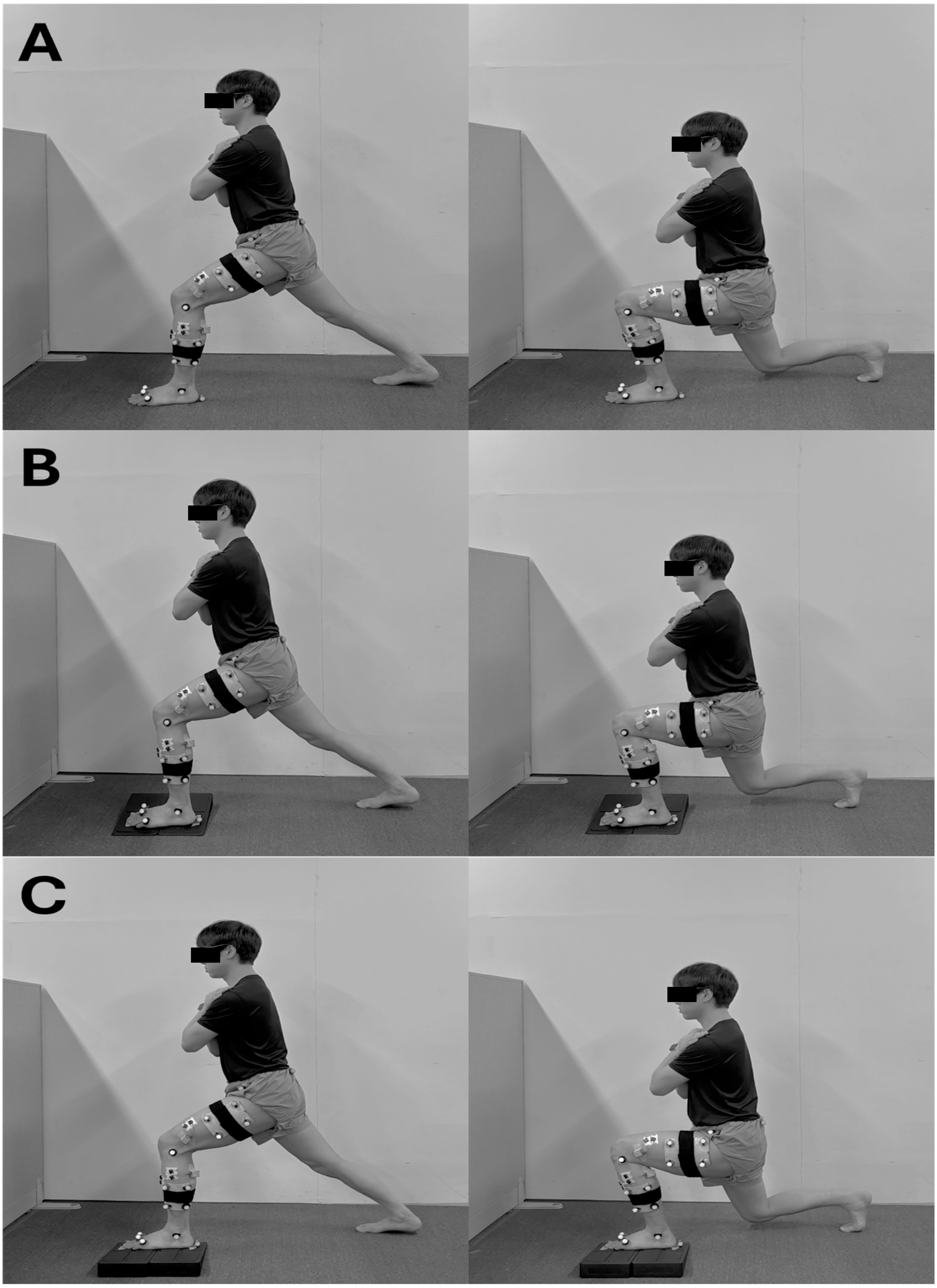

Supplementary Material — Approximate Pairwise Mean Differences with 95% Confidence Intervals

The following supplementary tables provide approximate mean differences (MD) with 95% confidence intervals (CI) for the pairwise contrasts (LW vs. MW, LW vs. NW, MW vs. NW) of the primary outcomes reported in Tables 2–4 of the main manuscript. Because the exact pairwise standard errors from the original SPSS output files were not retrievable, the CIs were reconstructed from the reported group means and standard deviations using a paired-sample approximation with t(0.025, 29) = 2.045. These intervals should be regarded as descriptive estimates intended to support quantitative interpretation, rather than fully calibrated inferential statistics. The Bonferroni-adjusted significance threshold for pairwise contrasts within each ANOVA family is $\alpha = 0.05/3 \approx 0.017$.

Table S1. Approximate pairwise mean differences (MD) with 95% confidence intervals (CI, in brackets) for muscle activation (%MVIC) during the descent and ascent phases of the split squat. Negative values indicate that the first condition was lower than the second.

Phase	Muscle	LW − MW	LW − NW	MW − NW
Descending	PL	+8.40 [+5.43, +11.37]	+9.44 [+7.43, +11.45]	+1.04 [−1.76, +3.84]
	TA	−5.98 [−8.45, −3.51]	+0.41 [−1.96, +2.78]	+6.39 [+3.63, +9.15]
	VL	−3.31 [−4.83, −1.79]	−0.77 [−2.11, +0.57]	+2.54 [+0.69, +4.39]
	VM	+3.74 [+2.38, +5.10]	+4.80 [+3.47, +6.13]	+1.06 [−0.19, +2.31]
Ascending	PL	+8.48 [+6.49, +10.47]	+8.68 [+6.59, +10.77]	+0.20 [−1.99, +2.39]
	TA	−8.58 [−9.05, −8.11]	+0.13 [−0.56, +0.82]	+8.71 [+8.16, +9.26]
	VL	−3.39 [−4.78, −2.00]	+1.55 [+0.29, +2.81]	+4.94 [+3.70, +6.18]
	VM	+4.85 [+3.70, +6.00]	+6.48 [+5.19, +7.77]	+1.63 [+0.39, +2.87]

Note. Values are in %MVIC. PL: peroneus longus; TA: tibialis anterior; VL: vastus lateralis; VM: vastus medialis; LW: lateral wedge; MW: medial wedge; NW: no wedge. Bonferroni-adjusted threshold for pairwise contrasts: α = 0.017.

Table S2. Approximate pairwise mean differences (MD) with 95% confidence intervals (CI, in brackets) for vertical ground reaction force (N/kg) during the descent and ascent phases of the split squat.

Phase	LW − MW	LW − NW	MW − NW
Descending	+0.57 [+0.36, +0.78]	+0.46 [+0.24, +0.68]	−0.11 [−0.34, +0.12]
Ascending	+0.75 [+0.56, +0.94]	+0.61 [+0.38, +0.84]	−0.14 [−0.34, +0.06]

Note. Values are vertical ground reaction force in N/kg. LW: lateral wedge; MW: medial wedge; NW: no wedge. Bonferroni-adjusted threshold for pairwise contrasts: $\alpha = 0.017$.

Table S3. Approximate pairwise mean differences (MD) with 95% confidence intervals (CI, in brackets) for sagittal-plane range of motion (in degrees) at the ankle, knee, and pelvis during the descent and ascent phases of the split squat.

Joint	Phase	LW − MW	LW − NW	MW − NW
Ankle	Descending	+3.88 [+2.52, +5.24]	+2.72 [+1.14, +4.30]	−1.16 [−2.40, +0.08]
	Ascending	+3.73 [+2.30, +5.16]	+2.25 [+0.82, +3.68]	−1.48 [−2.72, −0.24]
Knee	Descending	+3.44 [+0.80, +6.08]	+4.54 [+1.95, +7.13]	+1.10 [−1.46, +3.66]
	Ascending	+0.73 [−1.01, +2.47]	+2.45 [−0.18, +5.08]	+1.72 [−0.35, +3.79]
Pelvic	Descending	+1.88 [+1.19, +2.57]	+1.46 [+0.78, +2.14]	−0.42 [−1.10, +0.26]
	Ascending	+0.55 [−3.05, +4.15]	+0.09 [−2.78, +2.96]	−0.46 [−3.99, +3.07]

Note. Values are in degrees. LW: lateral wedge; MW: medial wedge; NW: no wedge. Bonferroni-adjusted threshold for pairwise contrasts: α = 0.017.
